# Building an immune-mediated coagulopathy consensus: early recognition and evaluation to enhance post-surgical patient safety

**DOI:** 10.1186/1754-9493-3-8

**Published:** 2009-05-22

**Authors:** Paul Ness, Michael Creer, George M Rodgers, Joseph J Naoum, Kenneth Renkens, Stacy A Voils, W Allan Alexander, the Recognition, Evaluation and Treatment of Acquired Coagulopathy Consensus (RETACC) Panel

**Affiliations:** 1 Division of Hematology, Johns Hopkins Medicine, 600 North Wolfe Street, Carnegie 667, Baltimore, Maryland 21287, USA; 2 Department of Pathology and Laboratory Medicine, St Louis University School of Medicine 1402 South Grand, St Louis, Missouri 63104, USA; 3 Division of Hematology, University of Utah Health Sciences Center, 50 North Medical Center Drive, Salt Lake City, Utah 84132, USA; 4 Division of Vascular Surgery, The Methodist Hosptial, 6560 Fannin Street, Suite 1006, Houston, Texas, 77030 USA; 5 8402 Harcourt Road, Suite 400, Indianapolis, Indiana 46260, USA; 6 Department of Pharmacy, Virginia Commonwealth University School of Pharmacy, 401 North 12th Street, PO Box 980042, Richmond, Virginia 23298-0042, USA; 7 BioSurgery and Medical Affairs, ZymoGenetics, Inc, 1201 Eastlake Ave E, Seattle, Washington 98102, USA

## Abstract

Topical hemostats, fibrin sealants, and surgical adhesives are regularly used in a variety of surgical procedures involving multiple disciplines. Generally, these adjuncts to surgical hemostasis are valuable means for improving wound visualization, reducing blood loss or adding tissue adherence; however, some of these agents are responsible for under-recognized adverse reactions and outcomes. Bovine thrombin, for example, is a topical hemostat with a long history of clinical application that is widely used alone or in combination with other hemostatic agents. Hematologists and coagulation experts are aware that these agents can lead to development of an immune-mediated coagulopathy (IMC). A paucity of data on the incidence of IMC contributes to under-recognition and leaves many surgeons unaware that this clinical entity, originating from normal immune responses to foreign antigen exposure, requires enhanced post-operative vigilance and judicious clinical judgment to achieve best outcomes.

Postoperative bleeding may result from issues such as loosened ties or clips or the occurrence of a coagulopathy due to hemodilution, vitamin K deficiency, disseminated intravascular coagulation (DIC) or post-transfusion, post-shock coagulopathic states. Other causes, such as liver disease, may be ruled out by a careful patient history and common pre-operative liver function tests. Less common are coagulopathies secondary to pathologic immune responses. Such coagulopathies include those that may result from inherent patient problems such as patients with an immune dysfunction related to systemic lupus erythrematosus (SLE) or lymphoma that can invoke antibodies against native coagulation factors. Medical interventions may also provoke antibody formation in the form of self-directed anti-coagulation factor antibodies, that result in problematic bleeding; it is these iatrogenic post-operative coagulopathies, including those associated with bovine thrombin exposure and its clinical context, that this panel was convened to address.

The RETACC panel's goal was to attain a logical consensus by reviewing the scientific evidence surrounding IMC and to make recommendations for the clinical recognition, diagnosis and evaluation, and clinical management of these complications. In light of the under-recognition and under-reporting of IMC, and given the associated morbidity, utilization of health care resources, and potential economic impact to hospitals, the panel engaged in a detailed review of peer-reviewed reports of bovine thrombin associated IMC. From that clinical knowledge base, recommendations were developed to guide clinicians in the recognition, diagnosis, and management of this challenging condition.

## Findings

### Recognizing Immune-Mediated Coagulopathy Associated with Bovine Thrombin

IMC is thought to be a relatively uncommon, under-recognized, and iatrogenic medical condition that can result from exposure to non-human coagulation proteins, such as porcine FVIII and bovine thrombin [1,2]. Beginning in 1989, coincident with the rising utilization of bovine thrombin preparations for topical surgical hemostasis, a number of case reports of post-surgical bovine thrombin associated IMC began to appear in the literature [3-35]. IMC occurring unexpectedly in post-surgical patients who were either exposed or re-exposed to bovine thrombin preparations have continued to be reported in nearly all surgical specialties, though cardiac, pediatric and orthopedic/neurosurgical case reports comprise the majority (Table 1). Interestingly, in the panel's literature review of 64 cases with known or presumed surgical exposure to topical bovine thrombin, there is a near equal representation of bleeding versus non-bleeding case presentations. Given that the majority of data on this condition is from case reports, the true incidence is unknown, but numerous reports have appeared in the literature since the year 2000 [3,6,7,11,17-19,22,24,25,27-29,31,36]. The true incidence of IMC associated with bovine thrombin remains unknown due to the lack of a clinical trial designed to answer that question. The result is a mixture of low awareness and confusion in the clinical community about the delayed onset of antibody formation and the challenges posed by masking due to coexistent coagulopathies (e.g., consumptive, dilutive, drug-induced, etc.).

**Table 1:** Reports of bleeding and non-bleeding IMC

	Bleeding Cases	Non-Bleeding Cases	Total^†^
Cardiac	21	19	40

Ortho/Neurosurgery	5	5	10

Others	6	8	14

^**† **^Pediatric cardiac cases accounted for 9 of the reported cardiac cases

Number of patients who presented with bleeding or laboratory abnormality among 64 cases that contained reports of a surgical setting where known or presumed bovine thrombin exposure occurred [3-35].

This panel's extensive review found that IMC always has a delayed onset following surgical procedures where bovine thrombin was used; none of the 61 cases reviewed (where time from exposure to clinical presentation was recorded), presented with symptoms or laboratory abnormalities earlier than 5 days following exposure (Table 2). The mean time to clinical presentation or laboratory abnormality (whichever was earlier) was 32 days, highlighting the potential for significant delay in onset and the potential for out of hospital risk. The most common time to presentation reported was 8 days (mode), while the median time to presentation was 11 days. Earlier clinical presentation in the 7 to 14 day time frame has most often been attributed to secondary exposure, where a second surgical exposure to a bovine thrombin preparation acts as a type of immunologic booster for antibody development [37,38]. Typically, primary exposure involves a more delayed clinical presentation due to the need for de novo maturation of the plasma B-cell antibody response. This obligatory time frame, dictated by the cellular and humoral mechanisms necessary for antibody formation, can delay diagnosis or even conceal the occurrence of these events from the surgeons employing these agents. The panel subscribe that with a proper index of suspicion, the resulting delays in recognition and treatment are preventable as are the increased morbidity, mortality, increased length of hospitalization and increased utilization of blood products that accompany this acquired disorder.

**Table 2:** Time from bovine thrombin exposure to clinical presentation of IMC

	Total (n = 61)
Mean (days)	32

Median (days)	11

Mode (days)	8

Standard Dev (days)	65.8

95% CI (days)	25–49

Range (days)	5–365

Summary statistics from review of 61 cases where time from known or presumed exposure were documented [3-10,12-18,20-28,30-35].

The evidence indicates that bovine thrombin related IMC results from antibodies that develop in response to bovine thrombin exposure and its associated proteins such as bovine factor V [18,31,39-41]. Bovine thrombin preparations have been reported to have in addition to bovine thrombin, bovine factors V, IX, and X as well as non-specific, immunoreactive proteins presumed to be protein fragments [42]. These antibodies may cross-react with human coagulation proteins, significantly interfering with the normal clotting cascade. Clinical sequelae range from individuals with abnormal prothrombin time (PT), activated partial thromboplastin time (aPTT) and thrombin time (TT) who are asymptomatic and at risk for bleeding [18,25,28,29,31] to anaphylaxis [36], hemorrhage [19,27], or other critical adverse events [17,19,27,43,44]. Despite prolonged efforts to improve manufacturing methods and remove contaminating proteins [18,38,40,41,45], there is no clinical evidence that bovine thrombin antigenicity has been reduced.

Most severe bleeding episodes occur following repeated exposure to bovine thrombin [34,46] and although history and documented use are often the best indication of previous bovine thrombin exposure, charts or pharmacy records can be incomplete. Accordingly, due to the widespread use of bovine thrombin in many different types of surgery, prior exposure can be difficult to determine in some patients. Even if documentation of prior exposure is available, it is impossible to predict whether antibodies will form or clinical sequelae will result. However, when presented with a patient with difficult to explain coagulopathy, a history that includes previous bovine thrombin exposure or procedures where it was likely used should raise suspicion of the potential for IMC as an etiology.

## Evaluation and diagnosis

IMC should be included in differential diagnosis when there is unexplained postoperative bleeding and/or hematoma, unexplained PT, aPTT or TT in the absence of bleeding, an exaggerated response to anticoagulants in the post-operative period, or bleeding unresponsive to conventional treatment. Whether caused by antibodies to factor V or thrombin, IMC following secondary exposure to bovine thrombin generally begins between 7 – 14 days after repeated exposure to bovine thrombin; in the cases reviewed by the panel no patient presented with laboratory or symptomatic IMC earlier than 5 days following exposure (Table 2). Thus, patients may be at risk for delayed post-operative bleeding, especially those who are hospitalized more than a week after surgery; complex post-surgical recovery is presumed to be a risk factor for a more severe bleeding presentation. In this review, the presence of cross-reacting antibodies to factor V accounted for over 85% (28/32) in both the bleeding cases as well as the cases presenting with laboratory abnormalities alone (Table 3). Patients presenting with laboratory abnormalities in the late post-operative period are at risk for spontaneous hemorrhage. Relatively asymptomatic coagulopathy in discharged patients may go unrecognized exposing them to the inherent hemorrhagic risks of an unrecognized bleeding disorder. Auto-directed antibodies can be persistent, extending over a period of months or years [6,8,31,43]. However, clinical presentation of IMC may occur 1 month or more after an initial bovine thrombin exposure [17,43]. With subsequent exposures, the appearance of antibodies, prolonged coagulation tests, and clinical complications can occur earlier, though no cases have been reported earlier than 5 days following re-exposure [46]. Therefore, bovine thrombin-associated IMC should be suspected when there is unexpected post-operative bleeding that is not explained by common causes of post-operative coagulopathy. It should also be suspected for post-surgical patients who have abnormal coagulation tests upon return for postoperative follow-up, management of a surgical complication or prior to another surgical procedure.

**Table 3:** Number of patients presenting with either anti- Factor IIa (thrombin) inhibitor, anti-Factor V (FV) inhibitor, or both

	Bleeding Cases N = 32	Non-Bleeding Cases N = 32	Total N = 64
Inhibitor Type	n (%)	n (%)	n (%)

FIIa	4 (13)	4 (12)	8 (12)

FV	17 (53)	15 (47)	32 (50)

FIIa and FV	11 (34)	13 (41)	24 (38)

Sorted by clinical presentation and contained reports of a surgical setting where known or presumed bovine thrombin exposure occurred [3-35].

**Figure 1:**
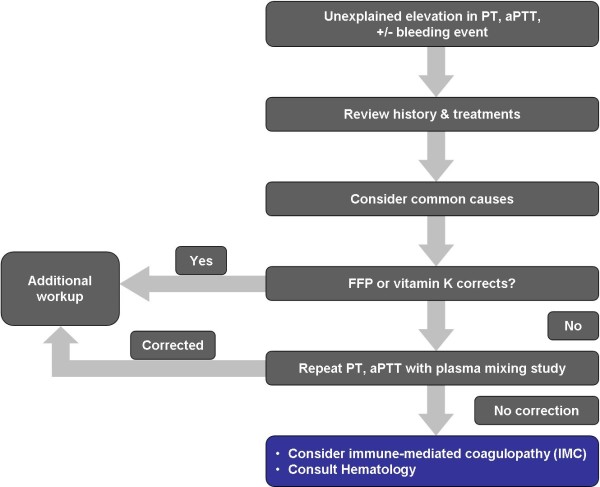
**Diagnosis of immune-mediated coagulopathy (IMC)**. In response to elevated PT/aPTT, IMC should be considered in the differential diagnosis for post-operative coagulopathy. Assays to rule out common causes of coagulopathies and mixing studies should be conducted. If the mixing study fails to correct, an inhibitor should be suspected. A hematology consult should be requested and factor assays conducted to confirm the diagnosis. (PT = prothrombin time; PTT = activated partial thromboplastin time; FFP = fresh frozen plasma).

The first step in evaluation and diagnosis of IMC is to measure PT and aPTT (Figure 1) [47]. If these tests are abnormal, common causes for post-surgical coagulopathy should be ruled out. These include surgical bleeding with hemodilution, DIC, vitamin K deficiency, and thrombocytopenia. In IMC, PT and aPTT will be persistently elevated as other common causes of post-surgical coagulopathy are eliminated. It should be noted that TT using human thrombin reagents may not be a reliable screening test and should not be used as a definitive determination of IMC. If IMC remains in the differential diagnosis after excluding other potential causes of acquired coagulopathy, consultation with a hematologist who is an expert in coagulation testing would be prudent.

In the presence of elevated PT and aPTT, a plasma mixing study should be ordered along with other laboratory studies (i.e., D-dimer, fibrinogen). Ideally, these studies should be ordered as soon as prolonged PT and aPTT are observed and before intervention of any kind, including administration of blood products. The clotting time of a 1:1 mix of patient plasma with normal pooled plasma should be evaluated immediately and at 60 and 120 minutes after incubation at 37°C. If the mixing study fails to correct and the clotting time falls outside of the reference range, then an inhibitor such as an antibody to a coagulation factor should be suspected. It should be noted that a mixing study is relatively insensitive for the early detection of a factor V inhibitor due to potentially low antibody titer levels in the period immediately following antigen exposure. However, if the PT and aPTT continue to be prolonged in the absence of a negative mixing test, the PT and aPTT should be monitored daily in conjunction with repeated mixing studies in any actively bleeding patient.

If not already initiated, a hematology and/or blood bank consult as well as confirmatory testing should follow a positive result from the mixing study. Laboratory evaluation should include specific quantitative assays of factor V and factor V inhibitor, if available. Most laboratories can evaluate factor V levels, but specific factor V antibody assays are not readily available and must be specially performed. More than one factor may be inhibited, but the majority of bovine thrombin-associated IMC cases reported in the literature have been linked to the development of pathogenic factor V inhibitors [17,19,27,31,43]. Transfusion will confound more specific factor and inhibitor assays; therefore, before initiating therapy additional blood samples should be obtained for more specific, definitive testing. Necessary supportive care and treatment should not be delayed while awaiting results since factor assay results may not be available for several days.

### Management of patients with an IMC

Management of patients with IMC may be challenging. While the following recommendations reflect the consensus reached by the panel, it is important to note that there is limited large-scale clinical evidence supporting these recommendations.

Patients can be divided into asymptomatic patients at risk for bleeding and actively bleeding patients. For the 25 cases involving non-bleeding (at risk) patients reviewed here, the most common interventions were prolonged hospitalization for observation, trials of FFP and vitamin K and in some cases immune-modulation with corticosteroids and/or intravenous immune globulin (IVIG) (Table 4). The panel supports some of these reported practices such that for the non-bleeding patient, vitamin K in conjunction with close observation is recommended. These patients should also avoid elective surgery until laboratory values normalize. The management of asymptomatic patients is largely patient-specific since the risk of spontaneous bleeding will vary as it does for patients on therapeutic anticoagulant regimens. Therefore, it is advisable that clinicians use their best judgment as to when to intervene. For patients with a history of comorbidities that increase their risk for bleeding, immunosuppression (e.g. corticosteroid therapy) may be reasonable. Prophylactic therapy to prevent spontaneous bleeding has been used to treat conditions like acquired hemophilia [48]. However, if surgery is planned, prophylaxis with the platelet transfusions necessary to treat the effects of anti-factor V antibodies entails risk and there is no definitive data demonstrating efficacy of this approach in otherwise asymptomatic IMC patients. Patient counseling, surveillance for complications, as well as serial factor assays and PT/aPTT assays, should be continued for six to eight weeks post-discharge or until values normalize. While the antibodies may be long-lasting in some patients [46], case reports suggest that the majority of the measurable inhibitors will eventually resolve. Parenthetically, skin testing for bovine thrombin induced hypersensitivity is not a useful screening technique because IMC is an IgG-mediated phenomenon while skin testing typically measures IgE-mediated mast cell degranulation and delayed T-cell mediated immune activation. Importantly, the duration of risk for an anamnestic response upon re-exposure is unknown, suggesting that re-exposure should be avoided and the patient counseled regarding bovine thrombin immunogenicity.

**Table 4:** Reports of resource utilization in non-bleeding patients with IMC

Category	Count	%
Extended LOS	23	92.0%

Vit K	9	36.0%

FFP	8	32.0%

IVIG	5	20.0%

Plasmapheresis	5	20.0%

Steroids	4	16.0%

PLT	1	4.0%

Chemotherapy	0	0.0%

RBC	0	0.0%

LOS – Length of stay; FFP – fresh frozen plasma; Vit K – vitamin K; IVIG – intravenous immune globulin; PLT – platelets; RBC – red blood cells

Interventions among the 25 non-bleeding patients for whom case reports contained documentation of managing immune-mediated coagulopathy [3-5,8,9,11,12,14,15,18,21,22,24-26,28-31,35].

Similarly, the management of actively bleeding IMC patients is highly speculative and patient specific. In the 18 reviewed cases involving patients bleeding at the time of clinical presentation, the most common reported interventions were prolonged hospitalization for supportive care, blood product transfusion, and vitamin K (Table 5). In 44% (8/18) of the cases, one or more interventions aimed at immune modulation occurred. These interventions included high dose corticosteroids, IVIG, plasmapheresis and potent chemotherapeutic immune suppression (cyclophosphamide, vincristine, others). Clearly, supportive care and replacement therapy with blood products are recommended. Fresh frozen plasma (FFP) and platelets should be administered in an effort to supplement coagulation factors in the face of a coagulopathy and to overcome the inhibitor [49]. Platelets may be particularly useful since normal platelets have factor V in alpha granules that is protected from circulating inhibitors (anti-factor antibodies). The immune system has a large capacity for antibody production. In situations where bleeding persists, further interventions designed to reduce circulating antibody titer may be indicated. Plasma exchange will accelerate inhibitor clearance and factor V replacement [27]. However, plasmapheresis only removes circulating antibody present in the intravascular space. Thus, due to rapid reappearance of antibody in plasma following redistribution from the larger extravascular space, the benefits of plasmapheresis may be only transient and multiple procedures will likely be required due to continued antibody production. In order to maintain factor V levels, plasma is indicated as the replacement fluid for plasmapheresis. In addition, there are inherent risks associated with plasma exchange and vascular access may be difficult to achieve in bleeding patients. Immunosuppressive therapy such as steroids, cyclophosphamide, rituximab, or IVIG (except during plasmapheresis), may be beneficial [17,19,28,29,31,50]. However, immunosuppression may take weeks to work. Therefore, immunosuppression alone is not sufficient for patients who are actively bleeding and may work best in conjunction with FFP and platelets. Because recombinant FVIIa (rFVIIa) depends upon an intact common coagulation pathway, rFVIIa is not recommended for treatment of actively bleeding IMC patients [17,43]. These recommendations are based upon a review of many case reports and not clinical trials, which would be difficult in view of the inherent difficulty in designing a trial where estimates of incidence rate vary widely.

**Table 5:** Reports of resource utilization in bleeding patients with IMC

Category	Count	%
Extended LOS	18	100.0%

FFP	12	66.7%

RBC	10	55.6%

Vit K	10	55.6%

Steroids	8	44.4%

PLT	8	44.4%

IVIG	7	38.9%

Plasmapheresis	5	27.8%

Chemotherapy	3	16.7%

LOS – Length of stay; FFP – fresh frozen plasma; Vit K – vitamin K; IVIG – intravenous immune globulin; PLT – platelets; RBC – red blood cells

Interventions among the 18 bleeding patients for whom case reports contained documentation of managing immune-mediated coagulopathy [6,7,9,10,15-17,20,23,26,27,30,31,33,34].

Once the patient has stopped bleeding, supportive care and follow-up should continue until antibodies resolve and PT/aPTT normalize. Further intervention may not be required once hemostasis is achieved and if no other surgical procedures are required. If the PT/aPTT is not resolving, a new mixing study and factor assays should be ordered. If the PT does not begin to normalize and the factor V level is not recovering and a risk of spontaneous hemorrhage remains, then immunosuppression with IVIG or steroids may be indicated. The risks of immunosuppression in the post-surgical patient should be carefully weighed. As with non-bleeding patients with IMC, post-hemorrhagic patients should be monitored until resolution of the inhibitor. In both asymptomatic and bleeding patients where bovine thrombin-associated IMC has been confirmed, patients should be counseled to avoid re-exposure to bovine thrombin due to the risk for an anamnestic (B-memory cell mediated) response. If further invasive procedures are planned, alternative topical hemostats should be considered.

## Conclusion

IMC is an iatrogenic and preventable medical condition that continues to cause patient morbidity and mortality. The panel's goal was to develop the recommendations included in this brief communication as a means of helping clinicians recognize, diagnose, and manage patients with IMC. The diagnosis of IMC requires clinical awareness and early re-evaluation of patient history; serial PT/aPTT tests, mixing studies, and factor assays are essential for the definitive diagnosis of this condition. While supportive care is largely patient specific, a number of management options are available including replacement transfusion, platelets, plasmapheresis, and immunosuppression. IMC is an avoidable condition and alternatives to bovine thrombin should be considered in patients with a history of unexplained post-operative bleeding. Avoiding the use of bovine thrombin preparations altogether would likely reduce the risk of bovine thrombin-associated IMC, thus avoiding the morbidity and extensive hospital resource utilization that are associated with this difficult to diagnose and manage condition.

## Competing interests

The RETACC panel acknowledges support from ZymoGenetics, Inc in facilitating the discussion of bovine thrombin-induced coagulopathy. The support allowed for the organization of a meeting for panel discussion and assistance with preparation and publication of the panel's recommendations.

## Authors' contributions

All authors contributed equally to the development of the manuscript and its revisions.
